# Bereavement and Resilience in Earthquake Affected Individuals: Serial Mediation through Psychological Distress and Hope

**DOI:** 10.1007/s11126-025-10165-3

**Published:** 2025-06-07

**Authors:** Begum Satici, Seher Merve Erus, Fatma Miray Benzer, M. Engin Deniz, Neslihan Tekgül, Seydi Ahmet Satici

**Affiliations:** Department of Psychological Counselling, Faculty of Education, Yildiz Technical University, 34220 Istanbul, Türkiye

**Keywords:** Earthquake survivors, Bereavement, Psychological resilience, Psychological distress, Hope

## Abstract

Natural disasters have existed as long as the world itself. Earthquakes are among the most common and destructive natural disasters. In earthquake-prone countries like Türkiye, which experienced the February 6 earthquake centered in Kahramanmaraş, conducting studies to mitigate the negative psychosocial effects following such disasters is of great social importance. This study investigated the effect of bereavement on psychological resilience, as well as the mediating roles of psychological distress and hope in earthquake-affected individuals. A total of 387 participants (318 females, 82.2%; 69 males, 17.8%) with an average age of 25.6 years completed scales assessing bereavement, psychological distress, hope and resilience. Structural equation modeling revealed that psychological distress and hope fully mediated the relationship between bereavement and resilience. Specifically, bereavement increased psychological distress and reduced hope, which in turn negatively affected resilience. These findings highlight the critical role of addressing distress and fostering hope to support resilience in earthquake-affected individuals.

## Introduction

Natural disasters have existed as long as humanity and are an inevitable part of life [44]. These events pose an international challenge, impacting both the psychological and physical health of individuals and societies, as well as their ability to manage daily activities [3].

The effects of earthquakes on society are observed worldwide, including in Türkiye [26]. Disruptions to daily life can have traumatic effects on both individuals and society [13]. Psychological traumas resulting from earthquakes can adversely affect individuals’ mental health throughout their lives [31]. Although reactions to traumatic events vary among individuals, the psychological responses that follow trauma, while often not permanent, can affect individuals for extended periods [47].

Resilience refers to the capacity to recover quickly from adversity [61]. In psychology, resilience refers to a set of personal qualities that help individuals succeed despite adversity [14]. It involves managing significant changes, distress, or risks effectively [32] and recovering quickly under adverse conditions [33]. Low psychological resilience is often linked to neurotic personality traits [42]. Individuals with low neuroticism tend to be calm and well-adjusted, whereas those with high neuroticism experience frequent stress, anxiety, and emotional instability [6]. Psychological resilience involves adapting to change through the interaction of risk and protective factors in response to stress-inducing events, such as natural disasters [27]. Studies in the literature have examined the psychological resilience of individuals affected by earthquakes [9, 11], Jing & Xu-Guang, 2008; [68, 72], as well as the relationship between bereavement and psychological resilience [57, 62, 63]. In this context, the need to investigate mediating variables in the relationship between psychological resilience and bereavement among individuals affected by earthquakes has emerged.

Economic difficulties, poor communication, and separations following an earthquake can reduce perceived support, with bereavement reactions manifesting as anxiety, stress, and depression [5]. Bereavement refers to the sense of loss brought about by death, as well as the psychological, physiological, and behavioral responses to this loss [54]. Individuals who survive an earthquake often experience intense stress due to the traumatic loss of loved ones, homes, and jobs [19]. In Sichuan, studies found that many survivors exhibited severe bereavement symptoms following the earthquake, highlighting the profound impact of traumatic losses on individuals [35]. Similarly, it was observed that survivors of the Wenchuan earthquake continued to grieve even seven years later [74]. Factors such as witnessing corpses, housing difficulties, and losing at least one relative in the earthquake significantly contribute to bereavement development [20]. Additionally, individuals with higher psychological resilience prior to a loss tend to exhibit fewer bereavement symptoms afterward [4]. This study posits that psychological distress and hope may help elucidate the relationship between bereavement and psychological resilience among earthquake survivors.

### Psychological Distress and Hope as the Mediators

Psychological distress brings long-term symptoms such as apathy, insomnia or excessive sleep, loss of appetite or overeating, reduced energy, and feelings of guilt [1]. Studies have identified a negative relationship between depression and psychological resilience [10, 28, 77, 78]. Additionally, research with individuals who experienced earthquakes found that depression scores had a significant negative impact on psychological resilience [21, 29]. Accordingly, psychological distress is expected to mediate the relationship between bereavement and psychological resilience among earthquake-affected individuals.

Hope is defined as an individual’s belief in achieving their goals and is associated with positive psychological outcomes [49]. Complementary to hope, psychological resilience is an individual’s capacity to adapt by maintaining mental balance through personal and social resources [30, 37, 73]. Previous studies have demonstrated the relationship between hope and psychological resilience [8, 52, 55]. This link was further confirmed by a recent meta-analysis by Wang et al. (69, 70. Moreover, studies with individuals affected by natural disasters concluded that hope and psychological resilience are related [24, 38]. Based on this, it is expected that psychological distress will serve as a mediating variable in the relationship between hope and psychological resilience among individuals affected by earthquakes.

### The Present Study

This study examined the variables of psychological distress, hope, bereavement, and psychological resilience. To date, no research has investigated these four variables together in individuals affected by earthquakes. The aim of this study was to explore the nature of the relationships between these variables. Türkiye, located in an earthquake-prone zone, frequently experiences high-intensity earthquakes [25]. On February 6, 2023, one of the largest earthquakes in history—the Kahramanmaraş earthquake—struck Türkiye, destroying thousands of houses, hospitals, hotels, mosques, and bridges across 11 provinces and resulting in over 50,000 casualties [17, 43]. Individuals who are vulnerable due to disasters often experience psychological symptoms such as depression, anxiety, and stress [5, 75]. Given that psychological resilience is defined as the ability to successfully overcome life’s challenges [51], it is hypothesized that psychological distress and hope may mediate the relationship between psychological resilience and bereavement among earthquake survivors. In line with this information, the research aims to provide guidance for psychosocial interventions designed to mitigate the negative impacts of earthquakes and similar catastrophic natural events on society. Based on the literature, the research questions are as follows:

RQ1: Does psychological distress mediate the relationship between bereavement and psychological resilience in individuals affected by earthquakes?

RQ2: Does hope mediate the relationship between bereavement and psychological resilience in individuals affected by earthquakes?

RQ3: Is there a serial mediation effect of psychological distress and hope on the relationship between bereavement and psychological resilience in individuals affected by earthquakes?

## Methods

### Participants and Procedure

The researchers shared the scale form with the individuals affected by the earthquake through Google Forms. Although participation was voluntary, participants were informed that they could withdraw from the study at any time. The sample included 318 female (82.2%) and 69 male (17.8%), with an average age of 25.6 years. Variables such as gender, economic status, relationship status, employment status, loss of relatives in the earthquake, and social support are presented in Table 1.

**Table 1:** Participants’ characteristic

Features	*N*	%
Gender		
* Female*	318	82.2
* Male*	69	17.8
Economic Status		
* Low*	60	15.5
* Medium*	307	79.3
* High*	20	5.2
Relationship Status		
* Single*	226	58.4
* Dating*	74	19.1
* Married*	87	22.5
Employment Status		
* Working*	128	33.1
* Non-working*	259	66.9
Social Support		
* None*	35	9
* Low*	79	20.4
* Medium*	138	35.7
* High*	135	34.9

#### Ethics

This research was conducted in accordance with the Declaration of Helsinki. Approval was obtained from Yildiz Technical University Ethics Committee before data collection (Report Number: 20250404537).

### Measures

#### Depression Anxiety Stress Scale-21

The Depression Anxiety Stress Scale-21 (DASS-21) was developed by Lovibond and Lovibond [39] and adapted into Turkish by Sarıçam [50]. The DASS-21 consists of three dimensions: depression (e.g., *Item 3: “I couldn’t seem to experience any positive feeling at all”*), anxiety (e.g., *Item 15:* “*I felt I was close to panic”*), and stress (e.g., *Item 12:* “*I found it difficult to relax”*), totaling 21 items. This is a 4-point Likert-type scale, scored from 0 (“Not at all applicable to me”) to 3 (“Completely applicable to me”). High total scores on the scale indicate an increase in psychological distress. The scale’s reliability analysis showed a Cronbach’s alpha internal consistency of 0.92 [50]. In this study, the Cronbach’s alpha internal consistency was calculated as 0.93.

#### Core Bereavement Items

The Turkish adaptation of the Core Bereavement Items, developed by Burnett et al. [7], was conducted by Selvi et al. [54]. This scale assesses the severity of bereavement in individuals who have lost a loved one, such as spouses, parents who have lost a child, or adults mourning the loss of a parent. The scale consists of 17 items across three sub-dimensions: images and thoughts, acute separation, and bereavement. Sample items include, “*Do thoughts about him/her come to your mind whether you want them to or not (Item 2)?”* and “*Are you intensely preoccupied with dreams and memories about him/her (Item 6)?*” The Scale employs a 4-point Likert scale (0 = never, 1 = sometimes, 2 = most of the time, 3 = constantly/always/every time). Higher scores indicate that bereavement is experienced more intensely [54]. In this study, the Cronbach’s alpha internal consistency of the scale was calculated to be 0.97.

#### Dispositional Hope Scale

The adaptation study of the Dispositional Hope Scale, developed by Snyder et al. [59], was conducted by Tarhan and Bacanlı [66]. The scale aims to assess the hope levels of individuals aged fifteen and older. It consists of 12 items, each evaluated using an 8-point Likert-type rating scale. The scale includes two sub-dimensions: alternative ways of thinking and acting thoughts, along with filler items that are not related to hope. Sample items include, “*I can think of many ways to het out of a jam (Item 1.)*”, “*There are lots of ways around any problem (Item 4).*”, and “*I meet the goals that I set for myself (Item 10).*”. A high score on the scale indicates that the individual has a high level of hope. The reliability analysis of the scale revealed a Cronbach’s alpha internal consistency of 0.86 [66]. In this study, the Cronbach’s alpha internal consistency of the scale was calculated to be 0.86.

#### Brief Psychological Resilience Scale

The Brief Psychological Resilience Scale, developed by Smith et al. [58], was adapted into Turkish by Doğan [18]. This scale is used to assess the psychological resilience levels of individuals. It is unidimensional and consists of a total of 6 items [e.g., “*I tend to bounce back quickly after hard times (Item 1*).” and “*It does not take me long to recover from a stressful event (Item 3).*”]. The scale employs a 5-point Likert-type rating system (1 = not at all appropriate – 5 = completely appropriate), with possible scores ranging from 6 to 30. Higher scores indicate greater psychological resilience. The reliability analysis of the scale revealed a Cronbach’s alpha internal consistency of 0.83 [18]. In this study, the Cronbach’s alpha internal consistency was calculated to be 0.87.

### Data Analysis

The IBM Social Science Statistics 2023 package was used to analyze the data. First, skewness and kurtosis coefficients were assessed to examine the normal distribution of the scores obtained from the Depression Anxiety Stress Scale-21 (DASS-21), Core Bereavement Items, Brief Psychological Resilience Scale, and Dispositional Hope Scale. The analyses conducted to reveal the relationships between psychological distress, bereavement, psychological resilience, and hope were performed using the two-stage structural equation model (SEM) proposed by Anderson and Gerbing [2] through the AMOS Graphics program. In using the two-stage structural equation model, the measurement model was tested first, followed by the hypothetical model. Model fit was evaluated using GFI, CFI, NFI, IFI, RFI, TLI, SRMR, and RMSEA values.

## Results

### Preliminary Analysis

Correlation findings for the relationships between psychological distress, bereavement, hope, and psychological resilience are presented in Table 2.

**Table 2:** Relationship between variables

Variables	1	2	3	Mean	SD	Skewness	Kurtosis	Cronbach α
1. Bereavement	-			14.57	12.92	.75	-.02	.97
2. Psychological Distress	.212^**^	-		18.83	11.06	.72	.16	.93
3. Hope	-.222^**^	-.442^**^	-	46.69	7.36	-.41	.53	.86
4. Psychological Resilience	-.177^**^	-.511^**^	.555^**^	18.09	4.40	-.02	-.09	.87

^**^p <.001

When examining Table 2, a significant negative relationship is observed between bereavement and psychological resilience (*r* = −0.177, *p* < 0.001). In contrast, a significant positive relationship exists between bereavement and psychological distress (*r* = 0.212, *p* < 0.001), as well as a significant negative relationship between bereavement and hope (*r* = −0.222, *p* < 0.001). Additionally, there is a significant negative relationship between psychological resilience and psychological distress (*r* = −0.511, *p* < 0.001), while a significant positive relationship is found between psychological resilience and hope (*r* = 0.555, *p* < 0.001).

### Structural Equation Modeling

After establishing the relationships between the variables, the measurement model—representing the first stage of the two-step structural equation modeling process—was tested. This model examined the relationships among psychological distress, bereavement, hope, and psychological resilience. It included four latent variables and ten observed variables associated with them.

The findings revealed that the factor loadings varied between 0.79 and 0.97, and each of these coefficients was statistically significant. It was concluded that all goodness-of-fit indices for the measurement model were at acceptable levels [*χ*^*2*^ (29, *N* = 387) = 110,93 *p* < 0.001; GFI = 0.948; CFI = 0.975; NFI = 0.966; IFI = 0.975; RFI = 0.948; TLI = 0.961; SRMR = 0.033; RMSEA = 0.086].

**Fig. 1:**
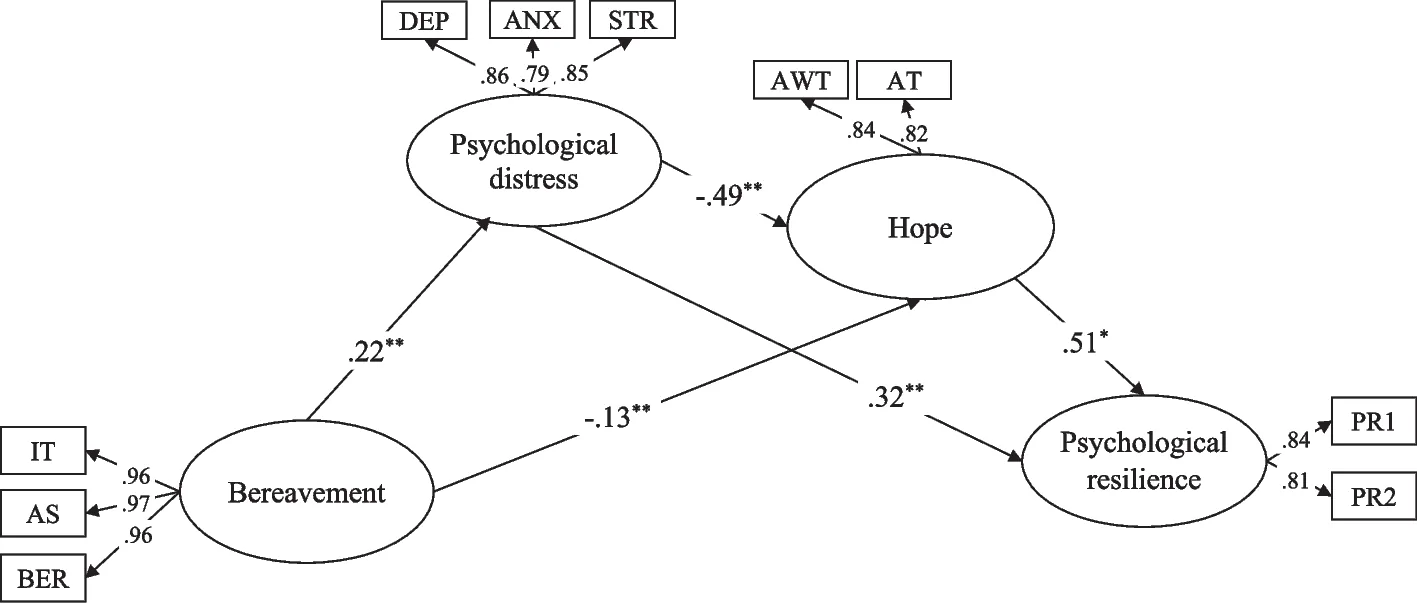
Structural equation modeling for the serial mediation model. Note. ^**^ *p* <.01, DEP: depression; ANX: anxiety; STR: stress; IT: images and thoughts; AS: acute separation; BER: bereavement; AWT: alternative ways of thinking; AT: acting thoughts; PRP: parcel of psychological resilience

After validating the measurement model, the hypothetical model was tested. Structural equation modeling was employed in the hypothetical model to investigate whether psychological distress and hope mediate the relationship between bereavement and psychological resilience. The model examined the relationship between bereavement and psychological resilience through the serial mediating roles of psychological distress and hope. The results indicated that all fit indices were at acceptable levels and that significant pathways were identified [*χ*^2^ (29, *N* = 387) = 155.64, *χ*^*2*^/df = 3.825; RMSEA = 0.08, SRMR = 0.033, TLI = 0.961, NFI = 0.966, CFI = 0.975, GFI = 0.948, RFI = 0.948, IFI = 0.975]. However, the path from bereavement to psychological resilience was found to be insignificant (*B* = 0.997, *p* > 0.05). Consequently, this pathway was eliminated, and a full mediation model was conducted. Good fit indices were obtained for the full mediation model, indicating that each path was significant (*χ*^*2*^ (29, *N* = 387) = 110.930, χ^2^/df = 3.698; RMSEA = 0.08, SRMR = 0.033, TLI = 0.963, GFI = 0.948, CFI = 0.975, NFI = 0.966, IFI = 0.975, RFI = 0.949). When comparing the partial and full models, the full mediation model was preferred since the direct path in the partial model was not significant. Figure 1 presents the findings of the serial mediation analyses.

To investigate the statistical significance of the mediating variables, 5,000 bootstrap samples were used. This method produced confidence intervals (CIs) with a 95% confidence level to examine indirect effects. Using this method, 95% of confidence intervals that did not include zero were considered statistically significant [23]. The relationship between bereavement and resilience was partially mediated by psychological distress, *b* = −0.030, *p* < 0.05, 95% BCa CI [−0.052, −0.014]. Additionally, the relationship between bereavement and resilience was partially mediated by hope, *b* = −0.123, *p* < 0.05, 95% BCa CI [−0.175, −0.083]. Finally, psychological distress and hope fully mediated the relationship between bereavement and resilience, *b* = −0.023, *p* < 0.05, 95% BCa CI [−0.042, −0.010] (Table 3).

**Table 3:** Standardized bootstrapping coefficients for the model

Model pathways	Coefficient	95% CI	
		Lower	Upper
Indirect Effect			
Bereavement → Psychological Distress → Hope → Psychological Resilience	-.023	-.042	-.010
Bereavement → Psychological Distress → Psychological Resilience	-.030	-.052	-.014
Bereavement → Hope → Psychological Resilience	-.123	-.175	-.083

## Discussion

People have faced and will continue to face natural disasters throughout their lives [44]. Natural disasters not only cause physical destruction but also deeply impact the mental health of individuals and societies [3]. Preparing for psychosocial interventions after an earthquake is particularly critical for countries located in earthquake-prone areas, such as Türkiye, which experienced a major earthquake centered in Kahramanmaraş on February 6. This event is considered one of the largest disasters in the world. This research aims to explore the effects of bereavement on the psychological resilience of individuals affected by earthquakes and to guide efforts to mitigate the negative psychosocial impacts that arise in society after devastating natural disasters like earthquakes. To this end, the study investigates the mediating effects of psychological distress and hope on the relationship between bereavement and psychological resilience in earthquake-affected individuals.

According to the first finding of this study, bereavement symptoms are positively associated with psychological distress levels in earthquake-affected individuals, which predicts psychological resilience. These findings support the hypothesis of RQ1. Bereavement is an emotional and psychological response to significant loss, with or without death [79]. Lindemann [36], who focuses particularly on the acute period of post-crisis bereavement, explains this experience through crisis theory, emphasizing that symptoms can occur immediately or later. Individuals experiencing bereavement may encounter physical discomfort, persistent thoughts about the loss, guilt for surviving, angry reactions, internalization of the lost person’s characteristics, and decreased functionality [48, 71]. Bereavement reactions include loss of appetite, emotional distance, guilt, anger, feelings of powerlessness, and exhaustion [36]. When examined, bereavement reactions are found to be similar to the characteristics of depression, such as reluctance, loss of appetite, feelings of guilt, powerlessness, and decreased energy [1]. For survivors of natural disasters, such events can become traumatic and trigger depression [67]. The literature includes studies that reveal the relationship between depression and bereavement [16, 34, 53, 65]. Additionally, characteristics such as calmness, hopefulness, optimism, and harmony [27] found in individuals with high psychological resilience are the opposite of the symptoms associated with bereavement and psychological distress. Research has also established a relationship between depression and psychological resilience [10, 22, 40, 69, 70]. In this context, the findings of this study align with the existing literature. The bereavement experienced by earthquake-affected individuals after material and/or moral losses leads to depressive symptoms, which subsequently negatively impact their psychological resilience. Consequently, bereavement in earthquake-affected individuals may increases psychological distress and decreases psychological resilience.

According to the second finding of this study, bereavement symptoms are negatively related to hope in earthquake-affected individuals, which in turn predicts psychological resilience. These findings support the hypothesis of RQ2. Hope refers to an individual’s belief in their ability to achieve their goals [45, 49]. It is multidimensional, dynamic, empowering, central to life, individual, and future-oriented [15]. For a bereaved individual, the loss may signify that their future is shattered and that they have lost their own goals [12]. According to hope theory, which focuses on goals that occupy consciousness, a hopeful individual can find pathways to their goals and utilize these paths with motivation [60]. Snyder [64] posits that people with low hope experience negative emotions and struggle to find alternative pathways, while those with high hope maintain positive emotions and are more flexible in pursuing their goals and exploring alternatives. In this framework, life’s challenges are viewed as obstacles to achieving desired goals, incorporating the individual’s objectives, emotional states, unexpected events, and stressors into the hope theory model [64]. When evaluating the contributions of the hope theory model to the literature concerning grieving individuals, it is evident that the losses experienced by those affected by earthquakes negatively impact hope as unexpected events and stressors. Unlike hope theory, which views hope as a focus on conscious goals [60], Lindemann’s [36] crisis theory highlights the intense cognitive reactions grieving individuals have toward their loss. Therefore, the decline in hope among grieving individuals further corroborates the findings of this research from another perspective. In a study conducted by Michael and Snyder [46] with individuals in the bereavement process, it was concluded that hope and psychological well-being are related, with hope playing a unique role in adapting to bereavement. Furthermore, several studies have revealed a relationship between hope and psychological resilience [52, 56, 76]. Notably, research on Ukrainian students affected by war also demonstrated a relationship between hope and psychological resilience [41]. Additionally, Ime [24] found a similar relationship in a study with earthquake survivors in Türkiye. In this context, the research findings align with the existing literature. The bereavement experienced by earthquake-affected individuals following material and/or moral loss diminishes their hope, ultimately negatively affecting their psychological resilience. As a result, bereavement in earthquake-affected individuals decreases psychological resilience by reducing hope.

According to the final finding of this study, bereavement symptoms are predicted by psychological distress and hope levels, which in turn predict psychological resilience in individuals affected by the earthquake. These findings support the hypothesis of RQ3. Thus, bereavement associated with high psychological distress and low hope indirectly predicts psychological resilience negatively. Moreover, the study concluded that psychological distress and hope fully mediate the relationship between bereavement and psychological resilience in earthquake-affected individuals. It was found that the psychological resilience of these individuals decreases as psychological distress increases and hope decreases. Earthquakes are among the most destructive and widespread natural disasters in the world (Erdoğan, 2023). For countries like Türkiye, which are situated in earthquake-prone zones, psychosocial interventions designed to enhance individuals’ psychological resilience following earthquakes should focus on reducing psychological distress and increasing hope while addressing bereavement.

## Implications

We believe that this research will contribute to reducing the negative effects of disasters on individuals in societies that experience devastating natural disasters, such as earthquakes. The findings obtained from this study may assist practitioners in designing interventions to strengthen psychological resilience by addressing bereavement, psychological distress, and hope. The findings obtained from this research may help in understanding the reasons behind the decrease in psychological resilience in individuals after an earthquake. Furthermore, this study could guide individual and group interventions aimed at increasing psychological resilience following an earthquake by focusing on bereavement, alleviating psychological distress symptoms, and establishing goals to foster hope. Researchers can build upon these findings to explore longitudinal changes in resilience after disasters, while policymakers may use the insights to develop community-based mental health initiatives that promote long-term recovery and resilience. In particular, therapists may use these findings to tailor trauma-informed approaches that target both emotional distress and hope-building in survivors. Post-disaster support programs can incorporate these elements into their protocols to enhance psychological recovery and resilience in affected populations.

## Limitations and Future Research

In addition to the findings of this study, there are several limitations. First, the sample predominantly consisted of young adults and females, which may limit the generalizability of the results to other age groups and genders. Additionally, the applicability of the model obtained in this study could be investigated in different cultural contexts. Longitudinal studies on psychological resilience could also be conducted to explore long-term changes in individuals affected by the earthquake. Longitudinal studies can reveal the cause and effect relationship. In addition, research can be conducted to determine the situations related to the specific loss situations of the participants (such as children and partners). Based on the findings of this study, post-earthquake psychosocial intervention programs can be developed.

## Conclusion

The bereavement resulting from the material and emotional losses experienced by individuals affected by the earthquake leads to symptoms of psychological distress and negatively impacts psychological resilience by diminishing the individual’s hope. Consequently, individuals with high levels of bereavement exhibit increased symptoms of psychological distress, which further reduces their hope. As a result, the psychological resilience of these individuals is also negatively affected.

## Data Availability

Data will be made available on request.

## Declarations

### Preregistration Statement

This study was not preregistered.
